# Iron accumulation in the substantia nigra is linked to functional network connectivity alterations in early-stage Parkinson’s Disease: an exploratory study

**DOI:** 10.1038/s41531-026-01400-0

**Published:** 2026-05-27

**Authors:** Benjamin C. Tendler, Giulia Serafica, Serena Turchi, Davide Risato, Konstantinos Koutsikos, Ilaria Parrotta, Moira Marizzoni, Annamaria Cattaneo, Elisa Mombelli, Samantha Saleri, Giulia Quattrini, Rita Barresi, Giorgia D’Este, Elena Pazienza, Mattia Spagna, Mattia Santin, Guido Caldarelli, Anna Vera Milner, Michele Gennuso, Leonardo Rigon, Nicola Filippini

**Affiliations:** 1 Centre for Integrative Neuroimaging, FMRIB, Nuffield Department of Clinical Neurosciences, University of Oxford, Oxford, UK; 2 IRCCS San Camillo Hospital, Venice, Italy; 3 Movement Control and Neuroplasticity Research Group, Leuven, Belgium; 4 Biological Psychiatry Unit, IRCCS Istituto Centro San Giovanni di Dio Fatebenefratelli, Brescia, Italy; 5 Department of Pharmacological and Biomolecular Sciences, University of Milan, Milan, Italy; 6 Department of Molecular Science and Nanosystems, Ca’ Foscari University of Venice, Venice, Italy; 7 Pathology Unit, Centro di Riferimento Oncologico di Aviano (C.R.O.) IRCCS, Aviano, Italy; 8 CNR-ISC, Consiglio Nazionale delle Ricerche - Istituto dei Sistemi Complessi, Rome, Italy; 9 Department of Neurology, Mirano Hospital, Mirano, Italy; 10 Department of Rehabilitation, Fondazione Camplani Casa di Cura “Ancelle della Carità”, Cremona, Italy; 11 Padova Neuroscience Center (PNC), University of Padova, Padova, Italy

**Keywords:** Biomarkers, Diseases, Neurology, Neuroscience

## Abstract

Parkinson’s disease (PD) involves dopaminergic nigrostriatal pathways degeneration and early structural brain abnormalities, including iron accumulation in the substantia nigra (SN). Understanding how these neurodegenerative changes relate to functional network adaptations may clarify disease progression and symptom variability. We used a cross-sectional design to evaluate resting-state functional MRI, quantitative susceptibility mapping (QSM) and structural MRI measures in 38 early-stage PD patients (Hoehn&Yahr ≤2; disease duration 4.4 ± 2.3 years) and 30 age- and sex-matched healthy controls. The SN showed the earliest increase in magnetic susceptibility in PD, indicating elevated iron levels, without detectable volumetric changes. Within the PD group, higher QSM SN susceptibility was associated with increased resting-state connectivity in motor, visual, dorsal-attention, and executive-control networks. Notably, connectivity within the SN-related executive-control network cluster correlated with better executive function and visuospatial memory. These findings highlight the sensitivity of QSM in early PD and the value of multimodal imaging for characterising early functional and cognitive consequences of PD.

## Introduction

Parkinson’s disease (PD) is the second most common type of progressive neurodegenerative disorder, affecting approximately 12 million people worldwide^1^. The prevalence of PD is increasing globally and it is projected to increase further^2^, with a recent UK estimate calculating a lifetime risk of diagnosis of 1 in 37^3^. A characteristic pathological hallmark of PD is the progressive loss of dopaminergic neurons in the substantia nigra (SN), a midbrain structure essential for dopaminergic signalling^4,5^. Early neuronal loss in the SN can precede clinical diagnosis based on motor symptomatology by years^6^, motivating the development of imaging markers sensitive to these neurodegenerative processes. Indeed, such measures would facilitate the identification of prodromal and/or early-stage PD before the full manifestation of classical motor and non-motor symptoms^7,8^, offering a vital window for early intervention and evaluation of novel neurotherapeutic targets. In the current therapeutic framework of PD, that increasingly aims at disease-modification, such in-vivo biomarkers would greatly facilitate patients’ stratification and treatment response tracking.

Advances in neuroimaging techniques, increased clinical awareness, and improved diagnostic criteria have shifted the average timepoint of PD diagnosis to earlier in the disease course^9–11^. When considering magnetic resonance imaging (MRI), structural and resting state functional MRI (rs-fMRI) techniques have provided important insights into the structural tissue damage and network-level brain alterations occurring in people with PD (pwPD)^12,13^. MRI studies examining volumetric differences in the SN between pwPD and healthy controls report reduced SN volume in pwPD, although results are less consistent at early stages^14^. Rs-fMRI studies have shown reduced functional connectivity within the sensorimotor network, relative to healthy controls^15^, alongside compensatory increases in premotor, cerebellar, executive networks in early PD, indicating that non-motor network functional reorganisation emerges at initial disease stages^16,17^. More recently, quantitative susceptibility mapping (QSM) has been proposed as a non-invasive imaging technique facilitating the indirect quantification of tissue iron through estimation of tissue magnetic susceptibility, which has been shown to be highly correlated with iron content in deep grey matter regions^18^. This is particularly relevant as iron accumulation in PD is considered a marker of pathology because of its strong association with dopaminergic neuronal loss, especially in the SN^19–21^. QSM-derived measures have been shown to have higher sensitivity and diagnostic performance than previous iron-sensitive methods (i.e. R2* mapping) for differentiating PD from controls, with sensitivity, specificity, and accuracy values ranging from 86 to 90%^22^. Moreover, QSM has also emerged as a promising tool for differentiating PD from atypical parkinsonian syndromes exhibiting distinct patterns of magnetic susceptibility in other brain regions^23^.

The integration of structural and functional imaging modalities could offer a more comprehensive understanding of *how* neurodegeneration propagates through large-scale brain networks, eventually leading to multidimensional (e.g. motor, cognitive, mood-related) clinical manifestations of PD. Moreover, modality integration may additionally enhance the specificity of PD markers and support the identification of mechanisms underlying network-level dysfunctions.

In this study, we use a multi-modal imaging protocol to investigate the relationship between QSM-derived estimates of magnetic susceptibility, structural MRI-derived volumetric measures and rs-fMRI-derived resting-state network (RSN) connectivity in a group of subjects with early-stage (Hoehn and Yahr ≤2; disease duration 4.4 years on average) PD. As QSM-related alterations in pwPD have been almost exclusively reported in the SN and basal ganglia (i.e. caudate, putamen, globus pallidum, thalamus)^24,25^, we focused on these regions for our investigation. Our study pursued two primary objectives. First, to determine which investigated brain regions exhibited increased magnetic susceptibility estimates or volumetric changes in pwPD compared to a group of healthy control (HC) participants. Second, to examine the relationship between QSM-derived metrics and RSNs strength of connectivity in pwPD using Independent Component Analysis (ICA), a data-driven approach for the unbiased identification of RSNs. The prior evidence implicating iron accumulation in the SN as a key contributor to PD^19–22^, raises the possibility that variations in SN iron may modulate resting-state functional connectivity. In this context, our work represents an initial attempt to link a candidate mechanistically relevant imaging biomarker with network-level functional alterations in PD, thereby complementing and extending previous findings. Consistent with this perspective, analogous approaches have been applied in other neurodegenerative disorders. For instance, in Alzheimer’s disease (AD), neurophysiological evidence indicates that the regional deposition of AD-related pathology, such as amyloid burden and tau accumulation, can influence large-scale functional connectivity patterns^26,27^. These findings provide a conceptual precedent for linking localised pathological burden to broader network-level alterations. Based on our findings, we additionally explored how imaging-derived measures of magnetic susceptibility and RSN connectivity strength related to neuropsychological performance in pwPD, with the aim of characterising early functional adaptations in pwPD.

## Results

### Study sample

Demographical and clinical characteristics of our study sample are summarised in Table 1. PwPD and HC did not differ for age, gender, years of education, handedness and body mass index (BMI). Mean age at PD diagnosis was 57.5 years old, with 28/38 pwPD (~75%) having less than 5 years of disease duration (mean 4.4 years). Males (57%) were more common than females (43%) among pwPD, in line with literature reports^28^. Mean Levodopa Equivalent Daily Dose (LEDD) was 490 mg and only one patient was treated with COMT inhibitors, reflecting a relatively modest dopaminergic treatment burden predominantly based on standard oral regimens. Mean MDS Unified Parkinson’s Disease Rating Scale (MDS-UPDRS) parts III and IV scores were 18.1 and 1.0, respectively. Twelve pwPD were classified in Hoehn and Yahr stage I, and twenty-six were in stage II.

**Table 1:** Sociodemographic and neuropsychological features of the 2 study groups

	HC (*N* = 30)	pwPD (*N* = 38)	*p*-value
Socio-demographics			
Age, y	64.5 (±10.9)	61.9 (±9.1)	0.29
Education, y	14.5 (±2.5)	13.2 (±3.1)	0.07
Gender (M/F)	13/17	22/16	0.34
Handedness (L/A/R)	2/1/27	0/3/35	0.21
BMI	23.6 (±3.3)	24.6 (±3.2)	0.24
Disease Duration, y		4.4 (±2.3)	
LEDD, mg		490.6 (±284.7)	
MDS-UPDRS, part 1		8.8 (±5.7)	
MDS-UPDRS, part 2		8.0 (±4.4)	
MDS-UPDRS, part 3		18.1 (±8.2)	
MDS-UPDRS, part 4		1.0 (±2.3)	
*Neuropsychological testing*			
MMSE	29.8 (±0.4)	29.7 (±0.6)	0.34
Phonemic Fluency	40.5 (±10.2)	44.9 (±11.3)	0.11
Semantic Fluency	50.2 (±11.3)	50.7 (±10.8)	0.84
Stroop, s	20.0 (±8.3)	21.8 (±14.4)	0.56
TMT B-A, s	60.8 (±29.5)	64.5 (±34.6)	0.64
Rey figure, immediate	33.2 (±2.1)	32.7 (±2.6)	0.43
Rey figure, delayed	18.2 (±4.4)	19.1 (±7.2)	0.58
RAVLT, immediate	56.2 (±9.2)	50.1 (±10.2)	0.01
RAVLT, delayed	12.9 (±2.3)	11.6 (±2.3)	0.02
Digit Span forward	5.7 (±1.1)	5.7 (±1.0)	0.88
Digit Span backwards	5.4 (±0.9)	5.2 (±1.1)	0.44
RAVEN	34.3 (±2.4)	34.3 (±2.1)	0.94
BDI	7.6 (±7.3)	10.8 (±8.9)	0.11

Values denote mean (±SD) or numbers of subjects.

*HC* healthy controls, *pwPD* people with Parkinson’s disease, *Y* years, *M* male, *F* female, *L* left-handed, *A* Ambidextrous, *R* right-handed, *BMI* body mass index, *LEDD* levodopa equivalent daily dose, *MG* milligrams, *MDS-UPDRS* movement disorders society unified Parkinson’s disease rating scale, *MMSE* mini mental state examination, *TMT B-A* trail making Test B-A score, *RAVLT* rey auditory verbal learning test, *BDI* Beck’s depression inventory.

### Neuropsychological evaluation

No statistically significant difference between pwPD and HC was observed for the majority of the tests adopted in our study. However, pwPD showed a significant reduction relative to HC for the Rey Auditory Verbal Learning Test (RAVLT) immediate and recall scores, in line with previous findings^29,30^. Notably, global cognition, assessed through the Mini Mental State Examination (MMSE), was overall preserved (mean scores [range]: pwPD 29.7 [28–30] vs. HC 29.8 [28–30]) and comparable among the two groups.

### MRI data results

#### T1-weighted structural scan

The Region-Of-Interest (ROI) analysis identified no group-related volumetric differences in the SN, the putamen, the caudate and the globus pallidum (See Table 2). However, a significantly reduced volume for the right thalamus and a trend towards significance for the left thalamus were found among pwPD relative to HC, in line with a previous study comparing ROI-extracted volumetric measurements in early stage pwPD relative to HC^31^. Following a Bonferroni multiple comparison correction (*p*-corrected <0.005), no regions remained statistically significant. Whole brain analysis using FSL-VBM showed no significant difference between the two study groups, supporting ROI-based results.

**Table 2:** Quantitative Susceptibility Mapping (QSM) derived magnetic susceptibility estimates and volumetric measurements from structural images of the two study groups

	Magnetic susceptibility values, ppm			Volumetry values, percentage		
	HC	pwPD	*p*	HC	pwPD	*p*
L Substantia Nigra	0.08 (±0.02)	0.10 (±0.04)	*0.02*	0.02 (±0.002)	0.02 (±0.003)	0.99
R Substantia Nigra	0.08 (±0.02)	0.10 (±0.03)	*0.002*	0.02 (±0.002)	0.02 (±0.003)	0.46
L Putamen	0.02 (±0.01)	0.02 (±0.02)	0.97	0.28 (±0.03)	0.27 (±0.03)	0.11
R Putamen	0.02 (±0.01)	0.02 (±0.02)	0.91	0.28 (±0.03)	0.26 (±0.03)	0.12
L Caudate	0.03 (±0.01)	0.03 (±0.02)	0.72	0.21 (±0.02)	0.19 (±0.02)	0.15
R Caudate	0.03 (±0.01)	0.03 (±0.02)	0.87	0.21 (±0.02)	0.21 (±0.02)	0.24
L Globus Pallidum	0.07 (±0.02)	0.08 (±0.02)	0.42	0.11 (±0.02)	0.11 (±0.03)	0.90
R Globus Pallidum	0.07 (±0.01)	0.07 (±0.02)	0.43	0.11 (±0.02)	0.11 (±0.03)	0.91
L Thalamus	−0.017 (±0.007)	−0.013 (±0.008)	0.07	0.47 (±0.03)	0.45 (±0.04)	0.08
R Thalamus	−0.016 (±0.007)	−0.012 (±0.007)	0.08	0.46 (±0.03)	0.44 (±0.04)	0.02

Values denote mean (±SD). QSM values were expressed as cerebral-spinal-fluid (CSF) referenced in units of parts per million (ppm), whereas volumetric values are expressed as percentage of whole-brain volume.

*L* left, *R* right, *HC* healthy controls, *pwPD* people with Parkinson’s Disease.

#### QSM

ROI analyses showed that pwPD had significantly greater magnetic susceptibility in both the left and the right SN relative to the HC group (Table 2), in line with a previous systematic review^32^. No significant differences were found in the other ROIs (Table 2). Following a Bonferroni multiple comparison correction (*p*-corrected <0.005), only the right SN reached statistical significance. Correlations between left and right SN QSM-derived values was highly significant (*r* = 0.84, *p* < 0.00001) and were averaged prior to comparison with RSN outputs.

#### Resting fMRI (rs-fMRI) scans

The pre-processing results of each of the thirty-eight pwPD were visually inspected to ensure registration accuracy. The optimal threshold for the use of FIX, to denoise functional images, was identified as 10 with a mean (median) true positive rate (TPR) and true negative rate (TNR) of 95.9% (100.0%) and 90.0% (92.2%), respectively. This is in line or above the suggested thresholding (TPR > 95%–TNR > 70%).

Multivariate Exploratory Linear Optimised Decomposition into Independent Components (MELODIC) identified twenty-five components representing group-averaged networks of brain regions with BOLD fMRI signals that were temporally correlated. The number of components was fixed to 25 based on an initial analysis of the population using model order estimation. The software generated 95 components resulting in an over-splitting of the commonly reported resting-state networks into several smaller components (as previously shown)^33^. Reducing the number of components automatically estimated by the software is therefore necessary in order to avoid the over-splitting of data. Previous studies with similar number of subjects as employed in our study used a similar approach^34,35^.

A total of 11 components were identified as true Resting-State-Networks (RSNs), whereas the remaining components mainly reflected motion and/or physiological artefacts and were therefore discarded. The 11 RSNs included: (1) medial visual network (MVN), (2) lateral visual networks (LVN), (3) salience network (SN), (4) dorsal-attention network (DAN), (5) default mode network (DMN), (6) motor network (MN), (7, 8) right and left frontoparietal networks (LFPN, RFPN respectively), (9) language network (LN), (10) executive control network (ECN) and (11) cerebellar network (CN) (Supplementary Fig. 1).

**Fig. 1:**
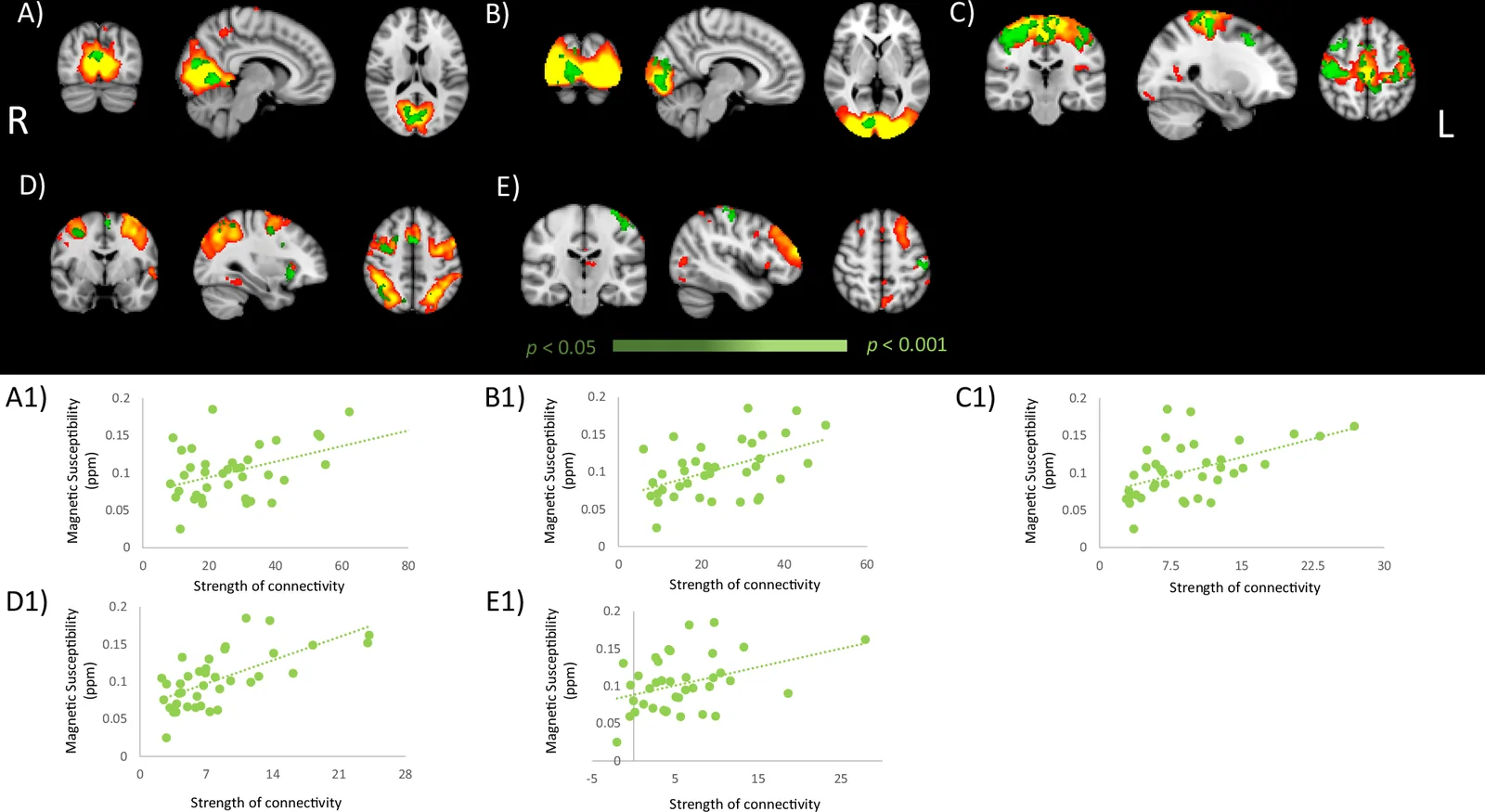
Brain clusters reflecting a significant positive correlation between magnetic susceptibility estimates in the substantia nigra (averaged across left and right hemispheres), as proxies of iron deposition, and functional connectivity measures (strength of connectivity) for pwPD in the following resting state networks. **A** Medial visual network, **B** lateral visual network, **C** motor network, **D** dorsal-attention network, **E** executive control network. Significant brain clusters are shown in green overlaid on the respective resting-state network. For the significant brain clusters, scatterplots are provided to illustrate the relationship between extracted brain region values from Resting State Networks: A1) Medial Visual Network, B1) Lateral Visual Network, C1) Motor Network, D1) Dorsal Attention Network, E1) Executive Control Network and magnetic susceptibility values. Results reported here are *p* < 0.05, corrected for multiple comparisons. R refers to the right and L to the left hemisphere.

Correlation analyses between RSNs and QSM-derived magnetic susceptibility measures in the SN revealed significantly positive correlation in five of the identified RSNs, namely the MN, the MVN, the LVN, the LFPN and the EFN (See Table 3 and Fig. 1). No significant clusters were identified for any RSNs showing a negative correlation between SN QSM-values and strength of connectivity. No correction for multiple comparisons across RSNs was applied in the present analyses, and results should therefore be interpreted with caution.

**Table 3:** Correlation cluster analysis between Resting State Networks (RSNs) and QSM-derived magnetic susceptibility measures in the Substantia Nigra

Resting state networks	Associated brain regions	Cluster size	MNI coordinates	T-max
Medial-visual network	Intracalcarine cortex	490	40-22-43	4.81
	Right lingual gyrus	440	40-33-36	5.29
LaterAl-visual Network	Right occipital cortex	1309	38-18-35	4.66
Motor network	Right precentral gyrus	3111	31-50-67	5.39
	Left precentral gyrus	834	64-50-67	4.33
Dorsal attention network	Right insular cortex	813	29-74-34	4.85
	Superior frontal cortex	357	42-68-65	5.17
	Right lateral occipital cortex	341	27-31-62	4.59
	Right precentral gyrus	248	29-61-60	4.34
Executive control network	Left postcentral gyrus	252	61-52-71	5.49

For each significant association, we report the associated brain regions, the cluster size (expressed in number of voxels), the coordinates of the peak in MNI space (in voxels) and the respective T-max value for the peak significant area within the reported cluster. Age, Disease Duration and Levodopa Equivalent Daily Dose were added as nuisance variables to exclude any potential confounding effects influencing our results. No correction for multiple comparisons across RSNs was applied.

### Correlation analyses between imaging-derived measures and neuropsychological tests and clinical measures

Correlation analyses using a two-tailed Pearson’s test between the QSM-derived SN values and the neuropsychological test scores within the pwPD group revealed no statistically significant associations (all *p* > 0.05). This suggests that iron accumulation in the SN, as indexed by tissue magnetic susceptibility, may not directly reflect cognitive performance as measured by standard neuropsychological assessments in our PD group. However, when we correlated the measure of connectivity strength extracted from the significant clusters, obtained investigating the relationship between SN QSM values and functional connectivity strength within resting-state networks, we observed meaningful correlations in the cognitive-related ECN network. Notably, the strength of connectivity within the SN-related clusters in the ECN (namely, the above reported the Left Postcentral gyrus) was significantly correlated with performance on neuropsychological measures of executive, inhibition and visuo-spatial memory functionality requiring a high executive function involvement. Specifically, time to complete the Stroop, Trail Making Test B and Trail Making Test B-A tests all revealed negative correlations (*r* = −0.382 *p* = 0.02, *r* = −0.4110 *p* = 0.01, *r* = −0.455 *p* = 0.004, respectively), meaning that the greater the connectivity strength score the quicker the time to complete the test. Conversely, performance at the Spatial Span Backward and Rey-Osterrieth Complex figure delayed recall revealed positive correlations (*r* = 0.396 *p* = 0.01, *r* = 0.393 *p* = 0.01, respectively), meaning that the greater the connectivity strength score the better the performance at the tests (scatterplots for the significant correlations are shown in Supplementary Fig. 2). *P*-values were not corrected for multiple comparisons, and these analyses were performed in an exploratory framework.

To assess whether the inclusion of motor regions in the ECN was specific to the pwPD group, analysis was performed in the HC group, revealing that premotor regions were not present within the network (Supplementary Fig. 3).

These findings suggest that whilst SN QSM values alone do not correlate with cognitive test performance, their association with functional connectivity, particularly in cognitive-related networks, may indirectly reflect the cognitive status in pwPD.

Correlation analyses between QSM-derived SN values and clinical measures from the MDS-UPDRS did not reveal any significant associations. Specifically, correlations between SN QSM values and MDS-UPDRS I, II, and III sub-scores were *r* = 0.12 (*p* = 0.47), *r* = 0.18 (*p* = 0.28), and *r* = 0.03 (*p* = 0.85), respectively. Similarly, voxel-wise correlation analyses between RSNs maps and MDS-UPDRS I, II and III sub-scores did not show any significant association.

## Discussion

This study provides novel insights into the pathophysiological underpinnings of PD by focusing on the accumulation of magnetic susceptibility changes, as measured by QSM, and its relationship with RSN connectivity. Our findings demonstrate that early-stage pwPD exhibit significantly increased magnetic susceptibility, indicative of iron accumulation, in the SN compared with age- and sex-matched HC. Importantly, this pathological alteration is detectable at relatively early disease stages, namely before the onset of motor complications or significant disability, and they may be observed even when conventional anatomical imaging does not show structural abnormalities. Moreover, we observed a positive association between SN magnetic susceptibility estimates and the strength of connectivity within several RSNs associated with both *sensory* (motor, medial-visual and lateral-visual) and *cognitive* (dorsal-attention and executive control) networks. Finally, we found that the strength of connectivity within the SN-related clusters in the ECN was significantly correlated with performance on neuropsychological scores demanding executive function involvement at various degrees.

Our observation that the SN is the region showing the most pronounced changes in magnetic susceptibility in early-stage PD aligns with the well-established neuropathology of PD, where dopaminergic neuronal loss and iron accumulation in the SN are hallmark features^36,37^. Our finding is in line with previous QSM studies across multiple cohorts, including early-stage or de novo PD, which have reported increased magnetic susceptibility in the SN even in patients with short disease duration^38,39^ and have suggested its potential as a longitudinal marker of disease progression^40^. Conversely, we did not detect any significant difference between pwPD and HC for the other deep brain areas examined. Findings in the literature for these structures are more heterogeneous, with studies reporting both significant alterations^39^ and others showing no detectable differences^22^ in early-stage pwPD relative to HC. These discrepancies may reflect methodological variability across studies, clinical heterogeneity, and the possibility that extranigral iron accumulation occurs later during disease progression. QSM is sensitive to iron content, and increased iron deposition has been implicated in oxidative stress, mitochondrial dysfunction, and neurodegeneration in PD^41^. The fact that QSM changes were detectable in the SN before structural changes reinforces the potential of QSM as an early biomarker for PD, providing a non-invasive approach to pathological iron accumulation monitoring. Early detection is clinically significant as it opens a window for intervention during the prodromal or early stages of PD, potentially before overt clinical symptoms fully develop. This issue is particularly critical in the current landscape of PD research, where a biological classification has recently been proposed as diagnostic criteria for research purposes^42^. Within this framework, iron-sensitive MRI techniques are considered candidate biomarkers for tracking disease state and progression. At the same time, substantial efforts are being devoted to the development of disease-modifying therapies. Consequently, the identification of early and reliable biomarkers is essential for accurately characterising patients enroled in clinical trials and for monitoring individual treatment responses. Expanding our understanding of the clinical correlates of non-ionising (and therefore safely repeatable) radiological biomarkers, such as SN-QSM, is therefore of critical importance. Future longitudinal studies in larger PD populations are warranted to determine whether early SN QSM increases predict clinical decline or conversion from prodromal/early stage to full PD.

A key finding of this study is the positive association between SN QSM values and RSN connectivity strength in networks subserving motor function (motor network), visual processing (medial and lateral visual networks), and higher cognitive functions (dorsal-attention and executive networks). This pattern suggests that iron accumulation in the SN may be linked with alterations in distributed brain networks that extend beyond the nigrostriatal motor circuit. While no formal correction for multiple comparison was applied across the 11 RSNs evaluated, we observed a highly consistent pattern of increased correlations with QSM-derived SN values across all networks, without any decreased or mixed effects. This uniform directionality provides additional confidence that the associations are meaningful and supports the notion that SN iron content may systematically influence functional connectivity in PD.

A possible interpretation of our results is that increased SN iron burden might induce compensatory increases in connectivity strength within these RSNs. Enhanced connectivity could reflect recruitment of additional neural resources or network reorganisation to maintain function despite underlying PD-related neurobiological changes. A similar effect has also been observed in participants with amnestic Mild Cognitive Impairment^43^. This condition often precedes the development of AD, thus representing its prodromal stage^44,45^. Early in the disease process, resting-state functional connectivity often follows an inverted U-shaped trajectory^46,47^. Indeed, functional connectivity within key networks, such as the default mode network (DMN), salience network, and frontoparietal network, all influenced by AD pathology, may initially increase, possibly reflecting a compensatory mechanism aimed at maintaining cognitive performance^46,47^. However, as the disease progresses, this early hyperconnectivity changes to declining connectivity, potentially due to network disruption and synaptic failure^48,49^. The combination of increased SN magnetic susceptibility and preserved or enhanced ECN functional connectivity may identify a subgroup of pwPD in a compensatory functional phase, distinct from individuals in whom similar iron burden is accompanied by network inefficiency.

Our findings align with prior neuroimaging studies reporting increased functional connectivity in early PD as a potential compensatory mechanism^50,51^. Moreover, a 4-year longitudinal magnetoencephalography study in early-stage PD showed initial increased alpha1-band connectivity which reversed over time and was associated with worsening of clinical and cognitive measures^52^. Understanding this trajectory is critical for identifying biomarkers of conversion and optimising the timing of therapeutic interventions. No voxel-wise correlation was observed between RSNs and motor symptoms (derived from the MDS-UPDRS) in our PD group. One possible explanation is that our PD cohort consisted primarily of ‘early-stage’ pwPD with minimal motor symptoms (we further discuss this point in the Limitations section). Additionally, imaging-derived markers may be associated with network alterations earlier and more sensitively than clinical symptoms, as shown in AD, where structural and functional network changes relate to amyloid and tau deposition long before diagnosis^53–56^, and similarly in PD, where resting-state connectivity may reflect early neuropathological changes even when symptoms are mild^57^.

The correlation between the SN-related cluster in the ECN (localised in the left precentral motor area) and neuropsychological measures may reflect a compensatory mechanism. Although the precentral motor area is typically considered part of the motor network rather than the ECN^58,59^, evidence shows it contributes to higher-order executive functions^60,61^. Recruitment of additional regions outside classical RSNs has been observed in neurodegenerative disorders, potentially reflecting compensatory processes, network reorganisation, or pathological spread^48,62^. Similar compensatory involvement of motor regions to support cognitive function has also been reported in aging and in pwPD^63,64^. We note that the cognitive analyses were not corrected for multiple comparisons. These findings should be considered exploratory and interpreted with caution.

Our investigation found no difference in SN volume between pwPD and HCs. Whilst MRI studies examining volumetric differences in the SN between pwPD and healthy controls have consistently report reduced SN volume in pwPD^14^, these findings have been found to be less consistent in earlier disease stage pwPD cohorts^65^, even when using ultra-high field scanners^66^. By contrast, neuromelanin-sensitive MRI sequence have demonstrated a significant decrease in the SN volume in pwPD compared to HCs even at an early disease stage, and may have been more sensitive to volumetric changes if utilised in our study^67^. Indeed, the neuromelanin-sensitive MRI sequence could have provided complementary information on SN dopaminergic neuron integrity and helped better contextualise the observed iron-related susceptibility changes. Future studies should examine the temporal relationship between susceptibility and volumetric changes to inform biomarker development.

Our volumetric analysis identified a statistically significant decrease in the volume of the right thalamus in pwPD, and a reduced volume in the left thalamus trending towards significance. The thalamus plays a key role in the progression of PD^68^. Whilst previous findings investigating changes of total thalamic volume in pwPD based on T1-weighted structural MRI acquisitions are inconsistent^31,69^, the volume of several thalamic nuclei has been recently linked to cognitive performance in pwPD^69^. It is notable that whilst our QSM analysis did not detect any statistically significant changes in magnetic susceptibility outside of the SN, the thalamus displayed the next most marked changes in magnetic susceptibility in pwPD (left/right thalamus *p*-value = 0.07/0.08, respectively). This suggests that QSM and structural MRI measures may be sensitive to PD-driven thalamic changes, motivating further analyses at the level of individual nuclei to identify how these changes manifest.

Several factors must be considered in interpreting these findings. Our study specifically involved early-stage, uncomplicated (not meeting 5-2-1 criteria for advanced PD nor treated with invasive therapies), pwPD characterised by relatively short disease duration (~4.5 years on average), limited motor functional impairment (Hoehn and Yahr ≤2) and dopaminergic treatment requirement (mean LEDD = 490 mg), and virtually absent motor fluctuations (mean MDS-UPDRS Part IV = 1.0). Additionally, cognitive performances were comparable to those reported in the age- and sex-matched HC group. This enabled us to provide an observational window on pathological changes happening in the early stages of PD. This may explain the lack of association between QSM-derived SN measures or RSNs functional connectivity and motor symptoms in our PD group, as the variability in imaging markers may not directly reflect the severity of clinical motor manifestations at this early stage of the disease. Furthermore, all assessments were conducted in the patients’ usual ON-medication state. Although this approach is intended to optimise tolerability and enhance the reliability of extended evaluations, particularly those involving cognitive and neuroimaging measures, it inherently influences the quantification of motor symptom severity. In line with the emerging concept of a biological definition of PD, establishing cohorts incorporating prodromal PD subjects^70^ (e.g. REM sleep behaviour disorder) and/or pwPD with a wider disease duration and motor/cognitive symptoms spectrum could clarify how magnetic susceptibility changes in the SN are associated with RSN’s functional connectivity changes, thus enhancing the predictive utility of QSM. In addition, whereas our association between imaging-derived measures and neuropsychological performance may suggest a compensatory mechanism linking increased SN susceptibility values and increased RSN strength of connectivity, other possibilities cannot be fully excluded. Indeed, it is possible that these connectivity alterations preceding measurable clinical decline may reflect maladaptive plasticity or even pathological hyperconnectivity (i.e. de-differentiation theory)^71^. Moreover, the SN was defined using an atlas-based segmentation rather than manually delineated subject-specific masks. Although atlas-based approaches provide improved reproducibility and reduce operator-dependent variability and have been used in prior PD imaging studies^72–74^, they may be less sensitive to individual anatomical changes associated with neurodegeneration. Notably, the rs-fMRI data were acquired with an eyes-closed protocol, which may increase the likelihood of participants falling asleep during scanning. Although the 10-minute acquisition ( ~ 600 volumes, TR = 1 s) provides a number of time points comparable to commonly used protocols and generally sufficient for reliable functional connectivity estimation, longer acquisitions may further improve the stability of correlation-based measures. Finally, the cross-sectional nature of the study restricts causal inference. Longitudinal designs are essential to determine whether SN QSM increases and RSN connectivity changes predict future clinical deterioration or reflect static disease features.

Our study provides further evidence that the SN is the earliest and most significantly affected region in terms of iron accumulation, as indicated from QSM in pwPD. We have demonstrated that this accumulation is associated with increased connectivity strength across several key resting-state networks involved in sensory and cognitive functions, suggesting that, at least in the early stage of PD, iron dysregulation in the SN may translate into distributed functional network adaptations that may help sustain largely preserved cognitive performance in early-stage PD. Investigating the association between SN QSM values and RSN connectivity in pwPD may provide a useful avenue for in-vivo biomarker exploration and pathophysiological research, refining prognostic and diagnostic approaches, and informing future therapeutic strategies, particularly in light of possible upcoming disease-modifying treatments.

## Methods

### Study sample

The study was approved by the local Ethics Committee (‘Comitato Etico per la Sperimentazione Clinica della Provincia di Venezia e dell’IRCCS San Camillo c/o Azienda ULSS 3 Serenissima’—Ethics Approval Number: 2022.13), and all participants provided written informed consent. The research was conducted in accordance with the ethical principles of the Declaration of Helsinki and Good Clinical Practice guidelines. Thirty-eight pwPD and thirty age- and sex-matched HC, involved in a wider project aimed at investigating the relationship between gut and inflammatory markers and brain changes (registered at ClinicalTrials.gov, Identifier: NCT05934188) were enroled for this study. PwPD eligible for this study were referred to the IRCCS San Camillo Hospital, Venice (Italy) from local healthcare institutions by the treating neurologists, experienced in the diagnosis and management of this condition. All assessments (clinical, neuropsychological and neuroradiological) were performed in the ON-medication state following the intake of the usual dopaminergic therapy.

Inclusion criteria for the pwPD cohort were: (1) PD diagnosis based on current Movement Disorders Society clinical criteria^75^; (2) optimised dopaminergic therapy stable for at least 6 months; (3) early-stage PD, defined considering both motor function [Hoehn and Yahr ≤2; absence of troublesome dyskinesia and/or fluctuations as defined by scores ≤2 in items 4.2 and 4.4 of the MDS-UPDRS^76^, respectively] and disease duration (<10 years since first diagnosis).

For all participants, socio-demographic information, such as age, sex, educational level, Body Mass Index (BMI) and handedness (assessed using the Edinburgh Handedness Inventory^77^) was collected. All pwPD underwent a thorough neurological evaluation performed by a movement disorders specialist (LR), comprising clinical assessment with the full MDS-UPDRS ([Part I (Non-motor experiences of daily living), Part II (Motor experiences of daily living), Part III (Motor examination) and Part IV (Motor complications)]) and recollection of the ongoing pharmacological therapy, including LEDD calculation^78^.

Exclusion criteria for all study participants were: (1) current or past history of other neurological or psychiatric disorders, (2) history of traumatic brain injury and/or brain vascular events (i.e. stroke or transient ischaemic attack) and/or neurosurgery (including Deep Brain Stimulation and Magnetic Resonance–guided Focused Ultrasound for the PD group), (3) substance abuse (including alcohol), (4) ongoing corticosteroid therapy, (5) contraindications to MRI scanning, (6) advanced PD stage according to the 5-2-1 criteria^79^ and/or requiring invasive treatment (including infusions/surgery), (7) significant cognitive impairment at screening evaluation as defined by MMSE < 26^80^.

### Neuropsychological evaluation

An extensive neuropsychological testing battery evaluating a wide range of cognitive and psychological domains was employed for this study. Reporting here the domains evaluated [in brackets the tests used]: *General cognitive health* [MMSE^81^]; *Attentive and Executive functioning* [Trail Making Test A and B^82^], and *Inhibition* [Stroop Test^83^]; *Abstract reasoning* [Raven PM 47^84^]; *Constructive and Visuo-spatial abilities* [Rey-Osterrieth Complex figure—copy (ROCF)^85^]; *Memory* – verbal short and long-term, verbal learning and spatial long term [Rey Auditory verbal Learning Test – Immediate and Delayed Recall^84^, Spatial Span Forward and Backward^86^, Rey-Osterrieth Complex figure – delayed recall (ROCF)^85^]; *Language* [Phonological fluency^84^, Semantic fluency^87^]; *Depression severity* [Beck’s Depression Inventory (BDI-II)^88^].

The total duration of the neuropsychological evaluation took approximately 60 minutes.

### MRI data acquisition

MRI scans were performed at the IRCCS San Camillo Hospital, Venice (Italy) using a 3 T Ingenia Scanner (Philips Inc., Amsterdam, Netherlands) with a 32-channel receive head coil. The neuroimaging protocol lasted approximately 45 min and is presented in Table 4. The T2-weighted FLAIR was acquired for clinical purposes only and visually inspected to exclude the presence of major vascular insults (i.e. stroke). During the rs-fMRI acquisition, participants were instructed to rest with their eyes shut and to try not to fall asleep. Earplugs and sponge pads were used to reduce scanning noise to protect the participants’ hearing and prevent head motion.

**Table 4:** MRI acquisition protocol

MRI Parameters	T1-weighted structural scan (MPRAGE)	*T2-weighted (FLAIR)*	*QSM (ME-GRE)*	rs-fMRI (Gradient-Echo EPI)
Resolution (mm^3^)	1.0 × 1.0 × 1.0	1.0 × 1.0 × 1.0	1.0 × 1.0 × 1.0	3.0 × 3.0 × 3.0
Field of view (mm)	240x240x180	240 × 240 × 180	224 × 224 × 140	168 × 240 × 240
TE (ms)	3.7	300	5.4, 10.6, 15.8, 21, 26.2, 31.4, 36.6	23
TR (ms)	8.1	5000	40	1000
Inversion Time (ms)	900	1700	NA	NA
Flip angle	8°	—	18°	50°
Parallel acquisition acc. factor	1 (AP), 2.3 (RL)	—	1 (FH), 2 (RL)	1 (AP), 2.3 (RL)
No. Volumes	—	—	—	600
EPI Factor	—	—	—	51
Acquisition time	4 min 37 s	5 min 15 s	10 min	10 min

*MPRAGE* magnetisation prepared rapid gradient echo, *FLAIR* fluid-attenuated inversion recovery, *QSM* quantitative susceptibility mapping, *ME-GRE* multi echo gradient echo, *rs-fMRI* resting-state fMRI, *EPI* echo planar imaging, *AP* anterior-posterior, *FH* foot-head, *RL* right-left.

### MRI data processing

#### T1-weighted structural scan

Following brain extraction (BET)^89^ and bias field correction^90^, structural volumes were co-registered to MNI space using both linear and non-linear (FLIRT and FNIRT, respectively)^91,92^ registration tools and tissue segmented into Grey Matter (GM), White Matter (WM) and Cerebrospinal Fluid (CSF) using FMRIB’s Automated Segmentation Tool (FAST)^90^.

An ROI analysis was performed in the SN and basal ganglia (caudate, putamen, globus pallidum, thalamus) to compare volumetric differences between the two study groups. SN masks were derived from the ATAG Atlas^93^ and registered into the participants’ native space. The left and right basal ganglia masks were obtained using FMRIB’s Integrated Registration and Segmentation Tool (FIRST), an automatic subcortical segmentation programme^94^. ROIs were visually inspected to ensure accuracy. Each participant GM ROI’s volume was normalised by the respective total brain volume to account for individual differences in head size.

A whole-brain Voxel-Based-Morphometry (VBM) analysis was performed to investigate cortical GM volumetric differences between HC and pwPD using FSL-VBM^95^ (default settings). Briefly, brain extracted and tissue-type segmented GM partial volume images (obtained using FAST^90^) were first aligned to standard space. The co-registered images were subsequently averaged, modulated, and smoothed with an isotropic Gaussian kernel of full-width at half-maximum (FWHM) of ~5 mm and registered to the GM study-specific template.

#### QSM

QSM was performed with the SEPIA toolbox^96^. Using coil-combined ME-GRE phase data exported from the scanner, phase data were first unwrapped using SEGUE^97^ with background field removal performed using vSHARP (radius 1–12 mm)^98^. Dipole inversion was performed using MEDI^99,100^ with automatic CSF referencing^101^.

As per structural images, ROI masks (generated from the structural MRI analysis) were registered into the native space of the QSM data for each subject and used to extract magnetic susceptibility estimates. These estimates were used to investigate differences between the two study groups.

#### Resting fMRI (rs-fMRI) scans

To account for susceptibility-induced distortions, we acquired two complementary Spin-Echo EPI volumes with reversed phase encodings and an identical readout and resolution to the rs-fMRI scan. Distortion correction was performed to the two SE-EPI volumes with TOPUP^102^ and subsequently applied to the rs-fMRI volumes prior to analysis. Pre-processing of rs-fMRI data was performed using first-level fMRI Expert Analysis Tool (FEAT) v.6.00^103^ and incorporated (a) motion correction, (b) brain extraction, (c) Gaussian kernel smoothing of FWHM of 5 mm, and (d) high-pass temporal filtering with a cut-off of 100 s (0.01 Hz). fMRI volumes were registered to an individual’s structural scan and standard space images with FLIRT and FNIRT and subsequently optimised using a boundary-based-registration approach^104^. FMRIB’s ICA-based X-noiseifier (FIX)^105^ was applied to denoise functional images from spurious signal and increase the possibility of identifying markers of effective connectivity.

Pre-processed and denoised functional data, containing 600 time points for each subject, were analysed using MELODIC^106^ from the PD group only, without inclusion of HC participants, to identify group-average RSNs. The ‘dual regression’ technique was employed to generate subject-specific versions of each of the group level spatial maps adopting the following steps: first, a spatial regression was performed by regressing the group-level ICA spatial maps onto each subject’s fMRI dataset. This step generated time-course matrices describing the temporal dynamics of each component for each subject. Next, a temporal regression was conducted using these time courses to estimate subject-specific spatial maps. Finally, the resulting component maps were combined across subjects into 4D files, enabling the assessment of between-subject or group-level differences^107^.

### Statistical analyses

Two-tailed T-test, Chi-Square/Yates’ correction and Pearson’s correlation were used to compare continuous and categorical socio-demographic, neuropsychological and ROI imaging-derived variables of the two study groups. Statistical analyses were performed using SPSS software (SPSS, Inc. version 28).

From the voxel-wise analysis of GM derived from VBM, between-subjects comparison (pwPD vs. HC), and correlation analyses between magnetic susceptibility values and RSNs strength of connectivity for pwPD rs-fMRI data were performed using a general linear model (GLM). For correlation analyses, QSM-derived values were included as covariate of interest, whereas age, disease duration and LEDD were added as nuisance (covariates of no interest) variables to exclude any potential confounding effects influencing our results. Voxel-wise GLM analysis was applied using Randomise, a permutation-based non-parametric testing (5000 permutations)^108^, and threshold-free-cluster-enhancement (TFCE) for clusters identification^109^. FWE corrected cluster significance of *p* < 0.05 and a minimum cluster size threshold of at least 200 contiguous voxels (to filter out potential noisy voxels)^110,111^ was applied to the suprathreshold clusters.

## Supplementary information

Supplementary Information

## Data Availability

The datasets generated and analysed during the current study are not publicly available due to Ethics Committee regulations regarding participant confidentiality, but are available from the corresponding author on reasonable request.
