# Supplementary material for: Iron accumulation in the substantia nigra is linked to functional network connectivity alterations in early-stage Parkinson’s Disease: an exploratory study
Source: NPJ Parkinsons Dis. 2026 May 27;12:197. doi: 10.1038/s41531-026-01400-0 (PMC13490446; doi:10.1038/s41531-026-01400-0)
Supplement: Supplementary file 1 [file 41531_2026_1400_MOESM1_ESM.pdf]

Supplementary Figure 1. Average group maps of Resting State Networks (RSNs) derived from pwPD. RSNs are shown superimposed on the MNI152 standard space template image. Red to yellow colours represent *Z* scores >=3. R refers to the right, L to the left hemisphere, S to superior, and I to inferior.

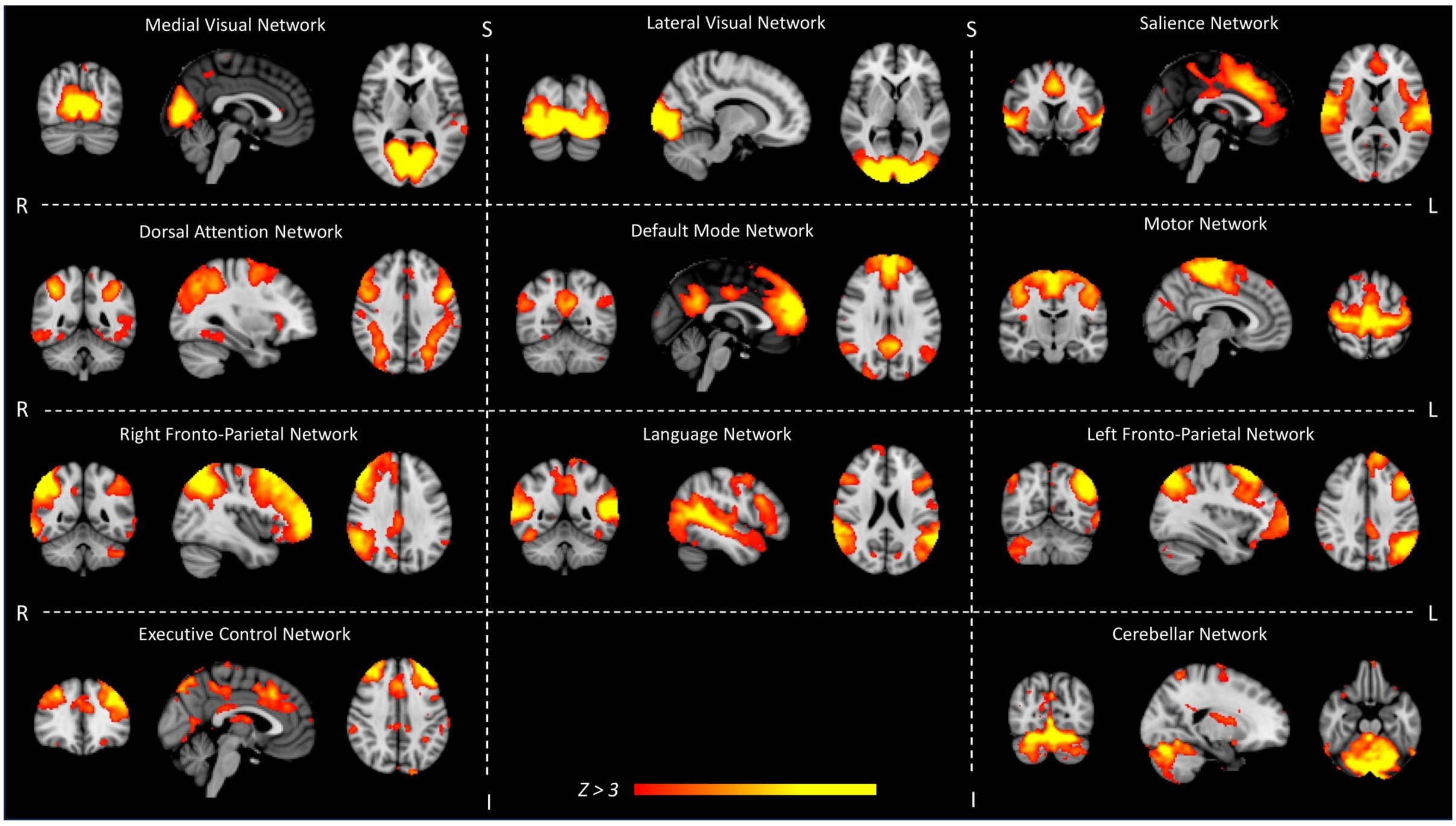

Supplementary Figure 2. Scatterplots showing the significant correlations between connectivity strength within the Substantia Nigra-related cluster of the Executive Control Network and the following neuropsychological measures: Stroop Test (r=-0.382 *p*=0.02), Trail Making Test (TMT) B (r=-0.4110 *p*=0.01), Trail Making Test (TMT) B-A (r=-0.455 *p*=0.004), Rey-Osterrieth Complex (ROC) figure delayed recall (r=0.393 *p*=0.01), and Spatial Span Backward (SSB) (r=0.396 *p*=0.01) scores. S=seconds.

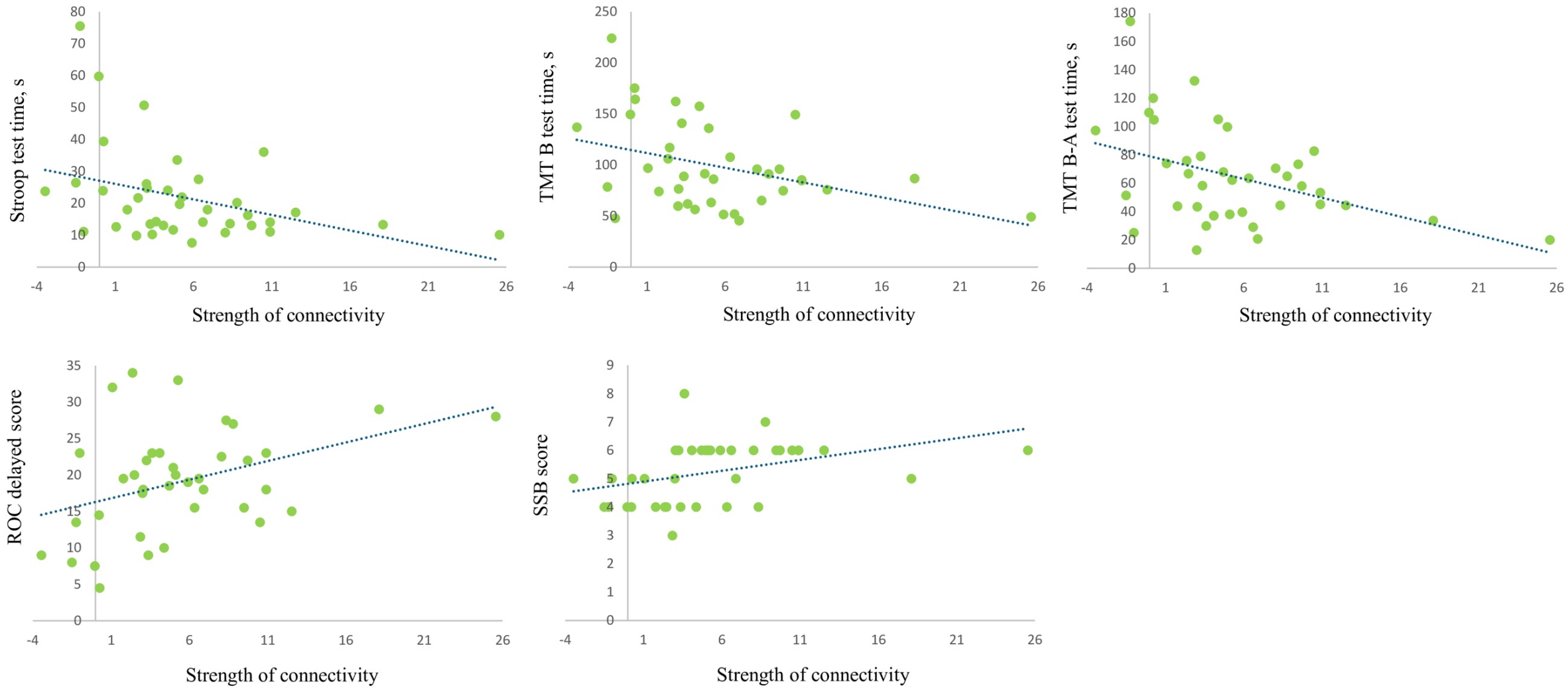

Supplementary Figure 3. Executive Control Network (ECN) group maps for the Healthy Control (HC) and Parkinson's disease (PD) groups (red–yellow). The left precentral cluster (green) corresponds to the region showing a significant positive correlation between substantia nigra magnetic susceptibility and functional connectivity in the PD group (see Figure 1 in the original manuscript). Mean connectivity strength values (±Standard Deviation) extracted from this cluster were close to zero in the HC group (−0.95±3.74) and higher in the PD group (6.84±5.49). R and L denote the right and left hemispheres, respectively.

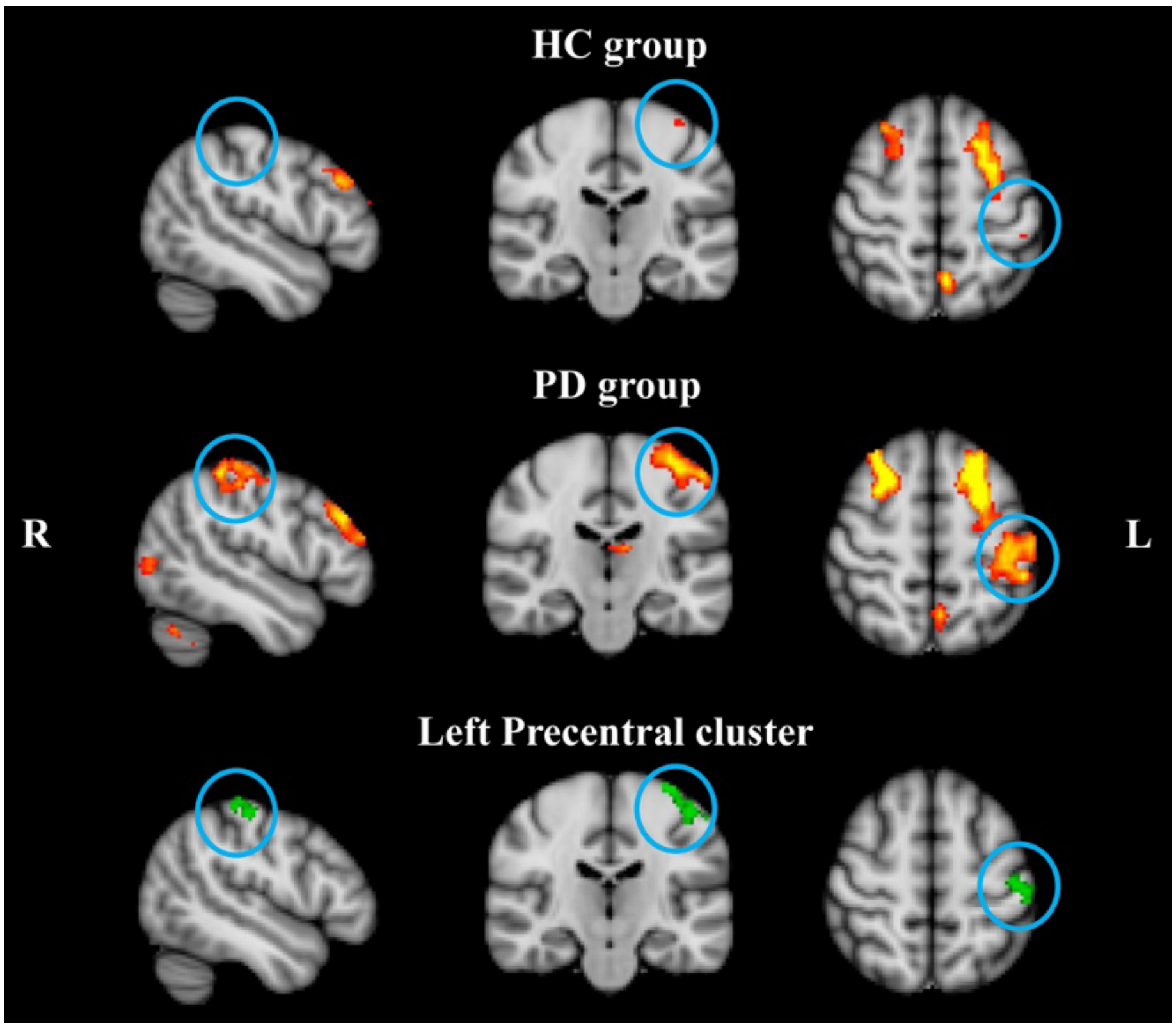

STROBE Statement—Checklist of items that should be included in reports of ***cross-sectional studies***

	Item No	Recommendation
**Title and abstract**	1	Indicate the study's design with a commonly used term in the title or the abstract<br><br>Cross-sectional ("We used a cross-sectional design to evaluate…")
		(*b*) Provide in the abstract an informative and balanced summary of what was done and what was found<br><br>In our abstract, we have reported a concise but explicative summary of our study sample, techniques and measurements used, main results and findings interpretation and usability.
**Introduction**		
Background/rationale	2	Explain the scientific background and rationale for the investigation being reported<br><br>Parkinson's disease is a progressive neurodegenerative disorder characterized by early dopaminergic neuronal loss and associated iron accumulation in the substantia nigra, which may precede clinical symptoms; however, the relationship between such pathological changes and large-scale brain network dysfunction remains unclear, motivating the use of integrated multimodal MRI approaches to identify sensitive in-vivo biomarkers and better understand early disease mechanisms.
Objectives	3	State specific objectives, including any prespecified hypotheses<br><br>The study aimed to identify brain regions showing altered magnetic susceptibility and volumetric differences in early-stage Parkinson's disease compared with healthy controls, and to examine the relationship between magnetic susceptibility (measured with Quantitative Susceptibility Mapping), particularly in the substantia nigra, and resting-state network connectivity, with the hypothesis that increased iron-related susceptibility would be associated with alterations in functional connectivity and neuropsychological performance.
**Methods**		
Study design	4	Present key elements of study design early in the paper<br><br>This observational cross-sectional study enrolled 38 patients with early-stage Parkinson's disease and 30 age- and sex-matched healthy controls to investigate differences in brain structure, magnetic susceptibility, and resting-state functional connectivity using a multimodal MRI protocol alongside clinical and neuropsychological assessments.
Setting	5	Describe the setting, locations, and relevant dates, including periods of recruitment, exposure, follow-up, and data collection<br><br>People with Parkinson's disease were recruited from local healthcare institutions and referred to the IRCCS San Camillo Hospital in Venice, as part of a wider study on gut, inflammatory, and brain markers (ClinicalTrials.gov Identifier: NCT05934188). All clinical, neuropsychological, and MRI assessments were conducted on-site.

Participants	6	(*a*) Give the eligibility criteria, and the sources and methods of selection of participants<br><br>People with Parkinson's disease were eligible if they met current Movement Disorders Society diagnostic criteria, had early-stage disease (Hoehn & Yahr ≤ 2, disease duration <10 years), and were on stable dopaminergic therapy for at least six months, whereas healthy controls were age- and sex-matched volunteers; participants were recruited through referrals from local neurologists to the IRCCS San Camillo Hospital in Venice, and all provided written informed consent. Full description of eligibility criteria can be found in the Methods section, Study sample sub-paragraph.
Variables	7	Clearly define all outcomes, exposures, predictors, potential confounders, and effect modifiers. Give diagnostic criteria, if applicable<br><br>The primary outcomes were MRI-derived measures of brain structure (volumetric differences in the substantia nigra and basal ganglia) and resting-state network connectivity, while the main exposure of interest was tissue iron accumulation estimated via quantitative susceptibility mapping (QSM). Predictors included disease status (Parkinson's disease vs. healthy control), and potential confounders such as age, disease duration, and dopaminergic medication dose (LEDD) were controlled for in analyses. Parkinson's disease' symptoms were derived from the Unified Parkinson's Disease Rating Scale.
Data sources/ measurement	8*	For each variable of interest, give sources of data and details of methods of assessment (measurement). Describe comparability of assessment methods if there is more than one group<br><br>Clinical and demographic data, including age, sex, educational level, body mass index (BMI), and handedness, were collected via standardized questionnaires and clinical interviews for both people with Parkinson's disease and healthy controls. Neuropsychological performance was assessed using validated tests covering cognitive, executive, memory, and mood domains. MRI-derived variables were obtained using a multimodal imaging protocol including T1-weighted structural scans for volumetry, quantitative susceptibility mapping (QSM) for iron content, and resting-state fMRI for network connectivity, with identical acquisition and processing procedures applied to all participants to ensure comparability across groups.
Bias	9	Describe any efforts to address potential sources of bias<br><br>To minimize selection bias, people with Parkinson's disease were recruited through referrals from experienced neurologists. Age- and sex-matched healthy controls were enrolled to avoid any potential socio-demographic confounding factor. All assessments were standardized across groups. MRI data were visually inspected and quality-controlled. Statistical analyses were controlled for potential confounders such as age, disease duration, and dopaminergic medication dose (LEDD) to reduce the impact of systematic differences between participants.
Study size	10	Explain how the study size was arrived at<br><br>The study sample of 38 people with Parkinson's disease and 30 healthy controls was determined based on availability within the broader ongoing project investigating gut, inflammatory, and brain

		markers (ClinicalTrials.gov Identifier: NCT05934188), aiming to recruit a balanced cohort sufficient for detecting group differences in multimodal MRI-derived measures while maintaining feasibility for extensive neuropsychological and imaging assessments.
Quantitative variables	11	Explain how quantitative variables were handled in the analyses. If applicable, describe which groupings were chosen and why<br><br>Quantitative variables, including MRI-derived volumetric measures, QSM-derived magnetic susceptibility values, and neuropsychological test scores, were analyzed as continuous variables, with structural ROI volumes normalized by total brain volume to account for individual differences in head size. With regard to group comparisons, participants were categorized into: A) people with Parkinson's disease and B) healthy control groups to examine disease-related differences, while covariates such as age, disease duration, and LEDD were included in regression models to control for potential confounding effects.
Statistical methods	12	(*a*) Describe all statistical methods, including those used to control for confounding<br><br>Continuous and categorical variables were compared between people with Parkinson's disease and healthy control groups using two-tailed t-tests and Chi-square tests (with Yates' correction, where appropriate), and correlations were assessed with Pearson's correlation coefficients. Voxel-wise analyses of gray matter volume and associations between QSM-derived magnetic susceptibility and resting-state network connectivity were conducted using general linear models, with age, disease duration, and Levodopa Equivalent Daily Dose (LEDD) included as nuisance covariates to control for potential confounding, and significance determined using permutation-based non-parametric testing with threshold-free cluster enhancement and family-wise error correction ($p < 0.05$).
		(*b*) Describe any methods used to examine subgroups and interactions<br><br>Subgroup analyses were conducted by examining region-specific MRI measures, including the substantia nigra and basal ganglia, and their relationship with resting-state network connectivity within the Parkinson's disease group using Independent Component Analysis (ICA) and dual regression. Interactions between QSM-derived magnetic susceptibility values and functional connectivity were assessed using voxel-wise general linear models, with age, disease duration, and LEDD included as covariates to account for potential modifying effects.
		(*c*) Explain how missing data were addressed<br><br>There were no missing data.
		(*d*) If applicable, describe analytical methods taking account of sampling strategy<br><br>Not applicable.
		(*e*) Describe any sensitivity analyses<br><br>Sensitivity analyses were performed by including age, disease duration, and Levodopa Equivalent Daily Dose (LEDD) as nuisance covariates in voxel-wise general linear models to assess whether the

		associations between QSM-derived magnetic susceptibility and resting-state network connectivity remained robust after controlling for potential confounding factors.
**Results**		
Participants	13*	(a) Report numbers of individuals at each stage of study—eg numbers potentially eligible, examined for eligibility, confirmed eligible, included in the study, completing follow-up, and analysed<br><br>A total of 38 people with early-stage Parkinson's disease and 30 age- and sex-matched healthy controls were included in the study. All participants completed clinical, neuropsychological, and MRI assessments, and data from all individuals including in the current manuscript were analyzed.
		(b) Give reasons for non-participation at each stage<br><br>There were no missing or excluded data for the participants included in the manuscript.
		(c) Consider use of a flow diagram<br><br>Not applicable.
Descriptive data	14*	(a) Give characteristics of study participants (eg demographic, clinical, social) and information on exposures and potential confounders<br><br>Full description of the socio-demographic and clinical info, and neuropsychological scores is provided in Table 1 of the manuscript. Very briefly, the study included 38 people with early-stage Parkinson's disease (mean age 61.9 years, 57% male, mean disease duration 4.4 years) and 30 healthy controls (mean age 64.5, 43% male), with no significant differences between groups in age, sex, educational level, handedness, or body mass index (BMI). People with Parkinson's disease had a mean Levodopa Equivalent Daily Dose of 490 mg, MDS-UPDRS Part I, II, III and IV scores of 8.8, 8.0, 18.1 and 1.0, respectively, representing a relatively mild clinical profile.
		(b) Indicate number of participants with missing data for each variable of interest
Outcome data	15*	Report numbers of outcome events or summary measures<br><br>Region-Of-Interest (ROI) analyses revealed significantly higher QSM-derived magnetic susceptibility in the left and right substantia nigra of people with Parkinson's disease relative to healthy controls, with the right substantia nigra remaining significant after Bonferroni correction. Volumetric measures showed a trend toward reduced right thalamus volume in people with Parkinson's disease, though no other regions differed significantly. Resting-state fMRI analyses identified 11 resting-state networks, of which five, namely the motor, medial and lateral visual, left frontoparietal, and executive control networks, showed significant positive correlations with substantia nigra QSM values. Neuropsychological correlations indicated that connectivity strength within executive control network clusters was associated with better performance on tests of executive function, inhibition, and visuospatial memory.
Main results	16	(*a*) Give unadjusted estimates and, if applicable, confounder-adjusted estimates and their precision (eg, 95% confidence interval). Make clear which confounders were adjusted for and why they were included

		Not applicable.
		(*b*) Report category boundaries when continuous variables were categorized<br><br>For group comparisons, participants were categorized into Parkinson's disease and healthy control groups, while volumetric ROI measures were normalized by total brain volume to account for individual differences in head size. No other continuous variables, such as QSM-derived magnetic susceptibility or neuropsychological test scores were categorized.
		(*c*) If relevant, consider translating estimates of relative risk into absolute risk for a meaningful time period<br><br>Not applicable for this study.
Other analyses	17	Report other analyses done—eg analyses of subgroups and interactions, and sensitivity analyses<br><br>Subgroup analyses within the Parkinson's disease group examined correlations between substantia nigra QSM values and resting-state network connectivity, revealing significant positive associations in five networks (motor, medial and lateral visual, left frontoparietal, and executive control networks). Further correlations between executive control network connectivity and neuropsychological test performance indicated that greater connectivity strength was associated with better executive and visuospatial function. Age, disease duration, and LEDD as covariates confirmed that these associations remained robust to potential confounding factors.
**Discussion**		
Key results	18	Summarise key results with reference to study objectives<br><br>In line with our study objectives (and with studies previously reported in the literature), we found that early-stage people with Parkinson's disease exhibit significantly increased magnetic susceptibility in the substantia nigra, indicative of iron accumulation, compared with age- and sex-matched healthy controls, while no consistent susceptibility differences differences were observed in other basal ganglia regions. Additionally, QSM-derived Substantia nigra values were positively associated with the strength of connectivity within multiple resting-state networks, including motor, visual, and executive control networks. Finally, cluster within the executive control network correlated with neuropsychological performance, suggesting that early Substantia nigra iron accumulation may influence distributed functional network adaptations in people with early Parkinson's disease.
Limitations	19	Discuss limitations of the study, taking into account sources of potential bias or imprecision. Discuss both direction and magnitude of any potential bias<br><br>This study has several limitations. Its cross-sectional design prevents causal inference, so it is unclear whether increased the substantia nigra magnetic susceptibility and altered resting-state network connectivity predict future clinical decline or reflect static disease features. The relatively small sample of early-stage people with mild Parkinson's disease may limit generalizability and reduce variability in clinical measures, potentially underestimating associations. All

		assessments were performed in the ON-medication state, which could attenuate motor symptom variability. The substantia nigra was defined using atlas-based segmentation rather than subject-specific masks, possibly reducing sensitivity to individual anatomical differences. The eyes-closed rs-fMRI protocol may have introduced noise if participants fell asleep, and no correction for multiple comparisons was applied across resting-state networks. Despite these limitations, the main finding that the substantia nigra iron accumulation is associated with functional network alterations in people with early Parkinson's disease remains robust.
Interpretation	20	Give a cautious overall interpretation of results considering objectives, limitations, multiplicity of analyses, results from similar studies, and other relevant evidence<br><br>Our findings indicate that early-stage people with Parkinson's disease shows increased substantia nigra iron accumulation detectable by QSM, which is associated with stronger connectivity in resting-state networks supporting motor, sensory, and cognitive functions. This aligns with prior studies suggesting early substantia nigra susceptibility changes and compensatory network adaptations in neurodegenerative disorders. However, the cross-sectional design, small sample size, limited clinical variability, and lack of multiple-comparison correction warrant cautious interpretation. While these associations may reflect compensatory mechanisms, alternative explanations, such as maladaptive hyperconnectivity, cannot be fully excluded. Overall, QSM and functional connectivity show promise as early Parkinson's disease biomarkers, but longitudinal studies in larger cohorts are needed to confirm their clinical relevance.
Generalisability	21	Discuss the generalisability (external validity) of the study results<br><br>The study's findings are primarily generalizable to early-stage, uncomplicated people with Parkinson's disease with relatively short disease duration, minimal motor impairment, and preserved cognitive function, similar to our cohort. Because all assessments were conducted in the ON-medication state, caution is warranted when applying these results to more advanced Parkinson's disease stages, patients with significant cognitive or motor deficits, or populations with different demographics or treatment regimens. Moreover, the relatively small sample size and cross-sectional design limit the ability to extrapolate findings to the broader Parkinson's disease population. Nonetheless, the observed substantia nigra QSM changes and RSN connectivity patterns provide useful insights into early pathophysiological mechanisms that may be relevant for similar early-stage Parkinson's disease cohorts.
**Other information**		
Funding	22	Give the source of funding and the role of the funders for the present study and, if applicable, for the original study on which the present article is based<br><br>Details of funding sources have been added to the "Acknowledgments" section. The main role of funders is related to support Researchers' salaries and data acquisition.

*Give information separately for exposed and unexposed groups.

Note: An Explanation and Elaboration article discusses each checklist item and gives methodological background and published examples of transparent reporting. The STROBE checklist is best used in conjunction with this article (freely available on the Web sites of PLoS Medicine at http://www.plosmedicine.org/, Annals of Internal Medicine at http://www.annals.org/, and Epidemiology at http://www.epidem.com/). Information on the STROBE Initiative is available at www.strobe-statement.org.
